# Analysis of factors affecting the prognosis of COVID-19 patients and viral shedding duration

**DOI:** 10.1017/S0950268820001399

**Published:** 2020-06-25

**Authors:** Jing Han, Li-xia Shi, Yi Xie, Yong-jin Zhang, Shu-ping Huang, Jian-guo Li, He-rong Wang, Shi-feng Shao

**Affiliations:** 1 Department of Medical Administration, Haihe Hospital, Tianjin University, Tianjin 300350, China; 2 Department of Prevention and infection management, Haihe Hospital, Tianjin University, Tianjin, 300350, China; 3 Department of Respiratory, Haihe Hospital, Tianjin University, Tianjin 300350, China; 4 Tianjin Institute of Respiratory Diseases, Tianjin 300350, China

**Keywords:** Clinical characteristics, COVID-19, duration of viral shedding, influencing factor, prognosis of disease

## Abstract

The clinical characteristics of patients with COVID-19 were analysed to determine the factors influencing the prognosis and virus shedding time to facilitate early detection of disease progression. Logistic regression analysis was used to explore the relationships among prognosis, clinical characteristics and laboratory indexes. The predictive value of this model was assessed with receiver operating characteristic curve analysis, calibration and internal validation. The viral shedding duration was calculated using the Kaplan–Meier method, and the prognostic factors were analysed by univariate log-rank analysis and the Cox proportional hazards model. A retrospective study was carried out with patients with COVID-19 in Tianjin, China. A total of 185 patients were included, 27 (14.59%) of whom were severely ill at the time of discharge and three (1.6%) of whom died. Our findings demonstrate that patients with an advanced age, diabetes, a low PaO_2_/FiO_2_ value and delayed treatment should be carefully monitored for disease progression to reduce the incidence of severe disease. Hypoproteinaemia and the fever duration warrant special attention. Timely interventions in symptomatic patients and a time from symptom onset to treatment <4 days can shorten the duration of viral shedding.

## Introduction

Novel coronavirus disease (COVID-19) can cause inflammation in the lungs, and the disease develops rapidly. As of 31 March, the severity and mortality rates of patients with COVID-19 in China were 23.25% and 4.06%, respectively [1]. A report from Wuhan showed that the mortality rates were 61.5% among critically ill patients with COVID-19 and 71% among those requiring mechanical ventilation [2]. During this pandemic, effective management of patients with severe COVID-19 is crucial to reduce mortality among the infected population. Viral shedding is one of the most important indicators of cure, and current reports have rarely analysed the duration of viral shedding. The clinical characteristics of 185 patients with COVID-19 diagnosed in Tianjin were analysed retrospectively to determine the factors affecting their prognoses and the duration of viral shedding with the aim of facilitating early treatment and improving patient prognosis.

## Materials and methods

### Patients

From 21 January to 8 May 2020, a retrospective study was conducted with all patients (⩾14 years) admitted to the hospital in Tianjin who were discharged after receiving a confirmed diagnosis of COVID-19. A total of 185 patients were finally included in this retrospective study.

### Data collection

Data were extracted from the hospital information system. Standardised forms were used to collect patients' clinical characteristics, including age, sex, comorbidities, former/current smoking, current drinking, the time from symptom onset to treatment, clinical symptoms, body temperature at admission, the PaO_2_/FiO_2_ ratio and complications. Imaging and laboratory examination results within 24 h of admission were collected, including CT examination results, routine blood examination results, C-reactive protein (CRP) levels, myoglobin (Myo) levels and other laboratory examination results.

### Outcomes

According to China's *Novel Coronavirus Pneumonia Diagnosis and Treatment Plan (Seventh Edition)* [3], the clinical classifications are mild, moderate, severe and critical.

Mild type: The clinical symptoms are mild, with no manifestations of pneumonia on imaging. Moderate type: Patients have fever, respiratory tract symptoms and other symptoms; imaging can show signs of pneumonia. Severe type: In adults, one of the following conditions must be met: (1) shortness of breath and a respiratory rate ⩾30 breaths/min; (2) oxygen saturation levels measured with a finger pulse oximeter at rest ⩽93%; or (3) PaO_2_/FiO_2_⩽300 mmHg. Critical type: One of the following conditions must be met: (1) respiratory failure requiring mechanical ventilation; (2) shock; or (3) extrapulmonary organ failure requiring intensive care unit monitoring and treatment.

Patients were divided into a good prognosis group and a poor prognosis group according to their clinical classification at discharge. Patients with mild and moderate COVID-19 were included in the good prognosis group, and patients with severe and critical COVID-19 and those who had died were included in the poor prognosis group.

### Discharge criteria

Patients who met the following conditions were discharged: (1) body temperature had returned to normal (<37.3 °C) and had remained normal for more than 3 days; (2) respiratory symptoms had improved significantly; (3) pulmonary imaging showed that acute exudative lesions had improved significantly; and (4) two consecutive nucleic acid tests on respiratory samples, such as sputum and nasopharyngeal swabs, were negative (a sampling interval of at least 24 h).

### Statistical analysis

The numerical variables with normal distributions are expressed as 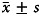, and comparisons between the two groups were performed with *t* tests. Continuous variables with non-normal distributions are represented as the median (quartile, *Q*), and non-parametric tests were used to compare the two groups. Count data are represented as *n* (%) and were compared with the *χ*^2^ test. Variables (age, diabetes, PaO_2_/FiO_2_ on admission, NLR and platelet count) with significant differences on univariate analysis and those with clinical credibility were included in a multiple factor regression [4]. Multivariate logistic regression was used to evaluate the risk factors associated with a poor COVID-19 prognosis. The results are presented as odds ratios (ORs) and 95% confidence intervals (CIs). To evaluate the discriminative performance of the logistic model, the area under the receiver operating characteristic (ROC) curve was calculated, comparing the actual outcome to the outcome predicted by the model.

In the univariate analysis, we used the log-rank test and Kaplan–Meier curve analysis for categorical variables and Cox regression analysis for continuous variables. The variables with *P* < 0.05 in the univariate analysis and variables with practical significance were included in the Cox proportional risk model, and the factors affecting the duration of viral shedding were identified through multivariate analysis.

## Results

### Epidemiological characteristics and hospitalisation data

The baseline characteristics of all 185 patients are shown in Table 1. A total of 16.2% of the 185 patients had a poor prognosis. Three non-survivors were included in the poor prognosis group. The mean age of the 185 patients was 44 ± 17.88 years, and 51.4% (95 patients) were male. The mean BMI of the patients was 24.61 ± 3.79 kg/m^2^. In this study, blood group B was the most common, accounting for 33.7% of the sample. Former/current smokers accounted for 12.4% (23 patients) of the patients, and 23.2% (43 patients) of the patients currently consumed alcohol. A total of 35.7% (66 patients) of the patients had one or more comorbidities, the most common of which were hypertension, diabetes and coronary heart disease (CHD). During hospitalisation, 9.2% (17 patients) of the patients had cardiac insufficiency, 31.9% (59 patients) of the patients had bacterial pneumonia and 12.4% (23 patients) of the patients had hypoproteinaemia. The median time from symptom onset to treatment was 4 (5) days.

**Table 1.** Univariate analysis of the severity of disease with regard to the prognosis of patients based on epidemiological characteristics and hospitalisation data

	Total population (*N* = 185)	Good prognosis group (*N* = 155)	Poor prognosis group (*N* = 30)	*P*-value
Age (years)	44 ± 17.88	40.6 ± 16.76	61.57 ± 12.42	<0.001*
Male-*n* (%)	95 (51.4%)	78 (50.3%)	17 (56.7%)	0.525
Blood type-*n* (%)				
A	60 (33.1%)	53 (34.9%)	7 (24.1%)	0.475
B	61 (33.7%)	52 (34.2%)	9 (31%)	
O	39 (21.5%)	30 (19.7%)	9 (31%)	
AB	21 (11.6%)	17 (11.2%)	4 (13.8%)	
BMI	24.61 ± 3.79	24.39 ± 3.99	25.76 ± 2.29	0.071
Former/current smoker-*n* (%)	23 (12.4%)	20 (12.9%)	3 (10%)	1
Current drinker-*n* (%)	43 (23.2%)	36 (23.2%)	7 (23.3%)	0.99
Comorbidities-*n* (%)	66 (35.7%)	44 (28.4%)	22 (73.3%)	<0.001*
Diabetes	28 (15.1%)	16 (10.3%)	12 (40%)	<0.001*
Hypertension	42 (22.7%)	27 (17.4%)	15 (50%)	<0.001*
Coronary heart disease (CHD)	16 (8.6%)	6 (3.9%)	10 (33.3%)	<0.001*
Cancer	3 (1.6%)	2 (1.3%)	1 (3.3%)	0.417
Cardiac insufficiency	17 (9.2%)	6 (3.9%)	11 (36.7%)	<0.001*
Hypoproteinaemia	23 (12.4%)	13 (8.4%)	10 (33.33%)	<0.001*
Bacterial pneumonia	59 (31.9%)	40 (25.8%)	19 (63.3%)	<0.001*
Time from symptom onset to treatment (days): *M(Q)*	4 (5)	4 (4)	6.5 (6)	0.037*

*P < 0.05.

A total of 185 patients were divided into two groups according to prognosis: 155 patients were in the good prognosis group, and 30 patients were in the poor prognosis group. Significant differences were identified between the two groups in terms of age (*P* < 0.001), comorbidities (*P* < 0.001), diabetes (*P* < 0.001), hypertension (*P* < 0.001), CHD (*P* < 0.001), cardiac insufficiency (*P* < 0.001), hypoproteinaemia (*P* < 0.001), bacterial pneumonia (*P* < 0.001) and the time from symptom onset to treatment (*P* < 0.05).

### Physical signs and laboratory examinations of the patients at admission

The physical signs and laboratory examinations of all 185 patients are shown in Table 2. Among the patients included in this study, 74.6% (138 patients) had fever, and 51.4% (95 patients) had cough, which are the common symptoms of COVID-19. The average temperature of the patients was 36.97 ± 0.81 °C. No significant difference in temperature was found between the two groups (*P* > 0.05). In total, 8.1% (15 patients) of the patients were asymptomatic, and most of them were classified as having had mild COVID-19 at discharge. The average PaO_2_/FiO_2_ ratio was 427.01 ± 171.05 mmHg. The value in the poor prognosis group was lower than that in the good prognosis group. A significant difference was detected between the two groups (*P* < 0.001). The median number of lung lobes involved in CT was 3 (4). The number of lung lobes involved in the poor prognosis group was significantly higher than that in the good prognosis group (*P* < 0.001). Significant differences were found between the two groups in terms of the lymphocyte count (*P* < 0.05), the NLR (*P* < 0.05), PLT (*P* < 0.05), and the levels of CRP (*P* < 0.05), Myo (*P* < 0.001) and D-dimer (*P* < 0.05) within 24 h of admission.

**Table 2.** Univariate analysis of the severity of disease with regard to the prognosis of patients based on the physical signs and laboratory examinations of patients at admission

	Total population (*N* = 185)	Good prognosis group (*N* = 155)	Poor prognosis group (*N* = 30)	*P*-value
Symptoms-*n* (%)	170 (91.9%)	141 (91%)	29 (96.7%)	0.295
Fever	138 (74.6%)	111 (71.6%)	27 (90%)	0.034*
Cough	95 (51.4%)	74 (47.7%)	21 (70%)	0.026*
Pharyngalgia	31 (16.8%)	28 (18.1%)	3 (10%)	0.279
Hypodynamia	40 (21.6%)	33 (21.3%)	7 (23.3%)	0.804
Diarrhoea	11 (5.9%)	9 (5.8%)	2 (6.7%)	0.855
Body temperature	36.97 ± 0.81	36.9 ± 0.77	37.3 ± 0.92	0.02*
PaO_2_/FiO_2_ on admission	427.01 ± 171.05	445.44 ± 176.28	331.74 ± 97.03	<0.001*
Number of lungs involved	3 (4)	3 (4)	5 (1.25)	<0.001*
Laboratory examinations				
WBC (109/l)	4.73 (1.98)	4.74 (1.9)	4.63 (2.23)	0.41
N (109/l)	3.04 (1.63)	2.99 (1.51)	3.09 (1.91)	0.773
L (109/l)	1.1 (0.69)	0.25 (0.13)	0.21 (0.15)	0.01*
NLR	2.70 (2.24)	2.61 (2.01)	3.73 (4.92)	0.024*
PLT (109/l)	187 (79.5)	194 (81)	157.5 (60.5)	0.005*
CRP (mg/l)	6.07 (28.76)	4.25 (22.21)	33.34 (47.29)	<0.001*
CK (U/l)	64 (56.5)	63 (53)	67.5 (185)	0.152
CK-MB (U/l)	7 (5)	7 (6)	7 (6)	0.315
LDH (U/l)	465 (194)	455 (160)	562.5 (334)	0.063
ALT (U/l)	34 (24)	35 (24)	29 (27)	0.078
Cr (μmol/l)	56 (25)	56 (23)	58 (25)	0.167
cTnI (ng/mL)	0.012 (0)	0.012 (0)	0.0125 (0.02)	0.2
Myo (μg/l)	27.9 (26.45)	28.75 (18.97)	50.1 (49.33)	<0.001*
D-Dimer (mg/l)	0.42 (0.47)	0.4 (0.47)	0.524 (0.65)	0.023*

WBC, white blood count cell; N, neutrophil; L, lymphocyte; NLR, neutrophil-to-lymphocyte ratio; PLT, platelet; CK, creatine kinase; MB Form(CK-MB), creatine kinase; LDH, lactate dehydrogenase; ALT, alanine aminotransferase; CRP, C-reactive protein; Cr, creatinine; cTnI, cardiac troponin I; Myo, Myoglobin.

*P < 0.05.

### Multivariate analysis of predictors of progression to severe disease

**Fig. 1.**
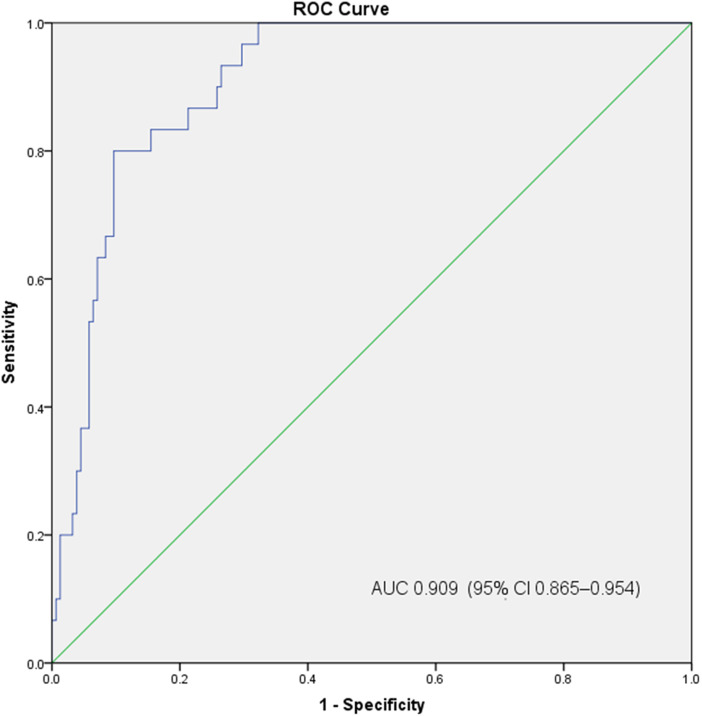
Receiver operating characteristic curve analysis of the prediction model for progression to severe disease, calculated by multivariate analysis.

In the multivariate regression analysis, age (OR = 1.089; 95% CI 1.046–1.133; *P* < 0.001), diabetes (OR = 3.311; 95% CI 1.093–10.031; *P* < 0.05), the time from symptom onset to treatment (OR = 1.185; 95% CI 1.042–1.347; *P* < 0.05) and PaO_2_/FiO_2_ (OR = 0.994; 95% CI 0.989–0.998; *P* < 0.05) were statistically significant (Table 3). We performed an ROC curve analysis with the variables identified in the multivariate analysis to predict progression to severe disease in patients with COVID-19. The model showed good discrimination (Fig. 1), with an area under the ROC curve of 0.909 (95% CI 0.865–0.954), suggesting that these variables can be used to predict a poor prognosis of the disease.

**Table 3.** Results of the univariate and multivariate analyses of the predictors of progression to severe disease, pooled estimates based on imputed data

Predictors	Univariate analysis				Multivariate analysis			
	OR	95% CI		*P*	OR	95% CI		*P*
		Lower	Upper			Lower	Upper	
Age (years)	1.083	1.05	1.117	<0.001*	1.089	1.046	1.133	<0.001*
Diabetes	5.792	2.366	14.176	<0.001*	3.311	1.093	10.031	0.034*
Time from symptom onset to treatment (days): *M(Q)*	1.102	1.015	1.196	0.037*	1.185	1.042	1.347	0.01*
PaO_2_/FiO_2_ on admission	0.994	0.99	0.997	<0.001*	0.994	0.989	0.998	0.01*
NLR	1.012	0.987	1.037	0.024*	0.968	0.921	1.016	0.19
PLT (109/l)	0.991	0.984	0.998	0.005*	0.992	0.983	1.001	0.083

OR, odds ratio; CI, confidence interval.

*P < 0.05.

### Analysis of outcome indexes and related factors in patients with COVID-19

The median duration of viral shedding after COVID-19 onset and hospitalisation was similar between the good and poor prognosis groups. The median duration of viral shedding was 17 (12) days from illness onset; the longest duration was 51 days, and the shortest duration was 4 days. The median duration of hospitalisation was 14 days. The median time to fever resolution was 3 days. The time to resolution of fever in the good prognosis group was 2 days, which was significantly shorter than that in the poor prognosis group (Table 4).

**Table 4.** Outcomes of patients in different prognosis groups

Outcomes	Total population (*N* = 182)	Good prognosis group (*N* = 155)	Poor prognosis group (*N* = 27)	*P*-value
Duration of viral shedding after COVID-19 onset, days	17 (12)	16 (13)	18 (13)	0.066
Time to fever resolution, days	3 (7)	2 (6)	7 (10)	<0.001*
Hospitalisation days, days	14 (11)	14 (11)	14 (10)	0.513

*P < 0.05.

**Fig. 2.**
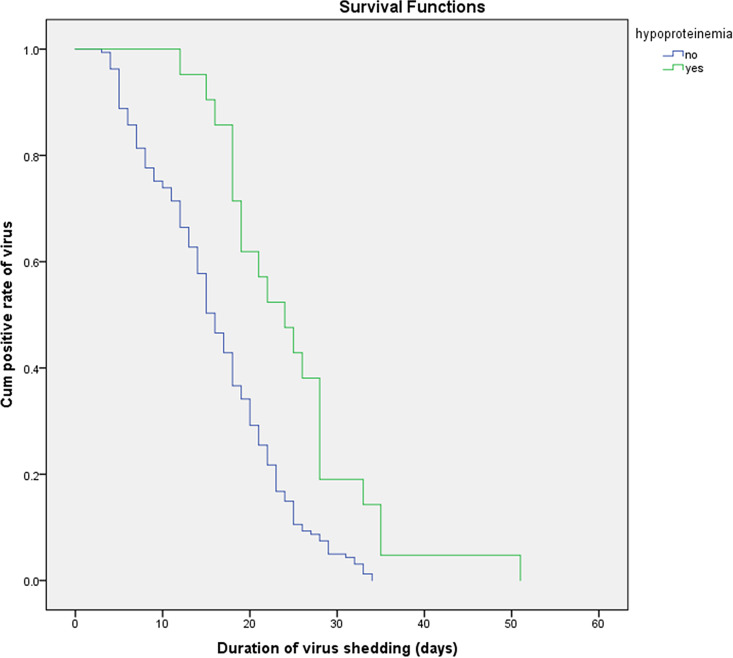
Kaplan–Meier plot for hypoproteinaemia.

**Fig. 3.**
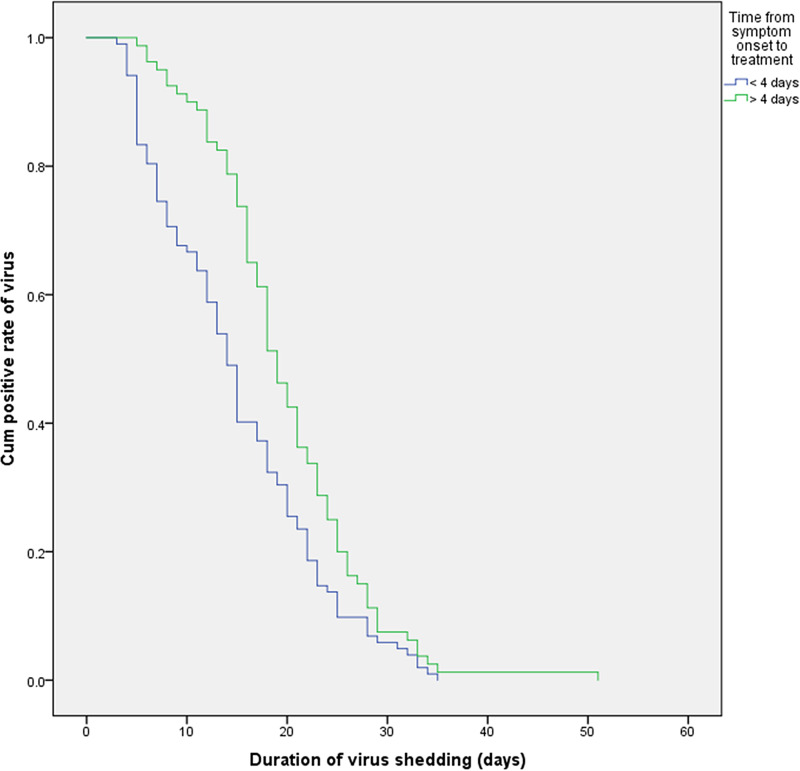
Kaplan–Meier plot for the time from symptom onset to treatment.

**Fig. 4.**
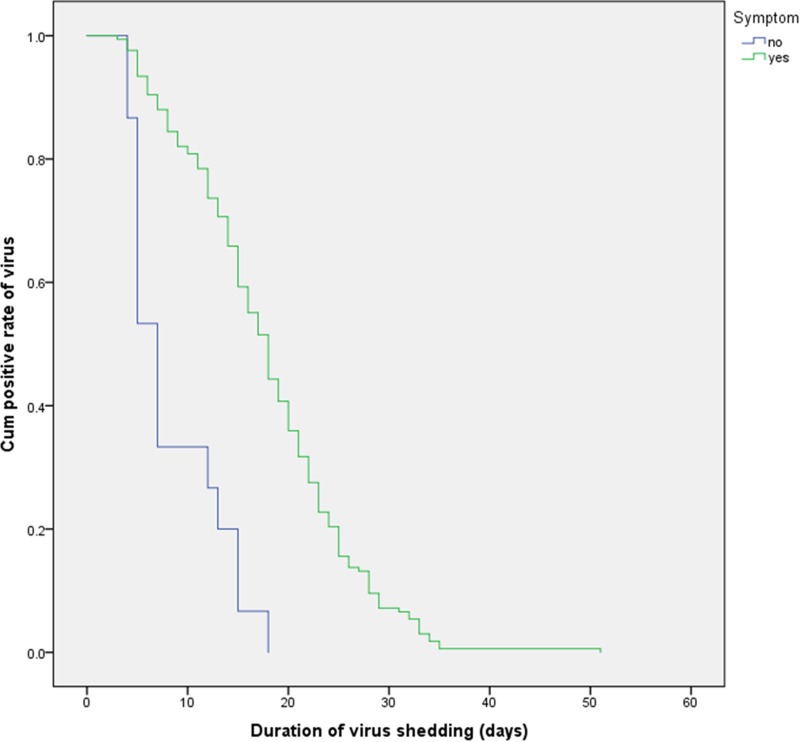
Kaplan–Meier plot for symptoms.

**Fig. 5.**
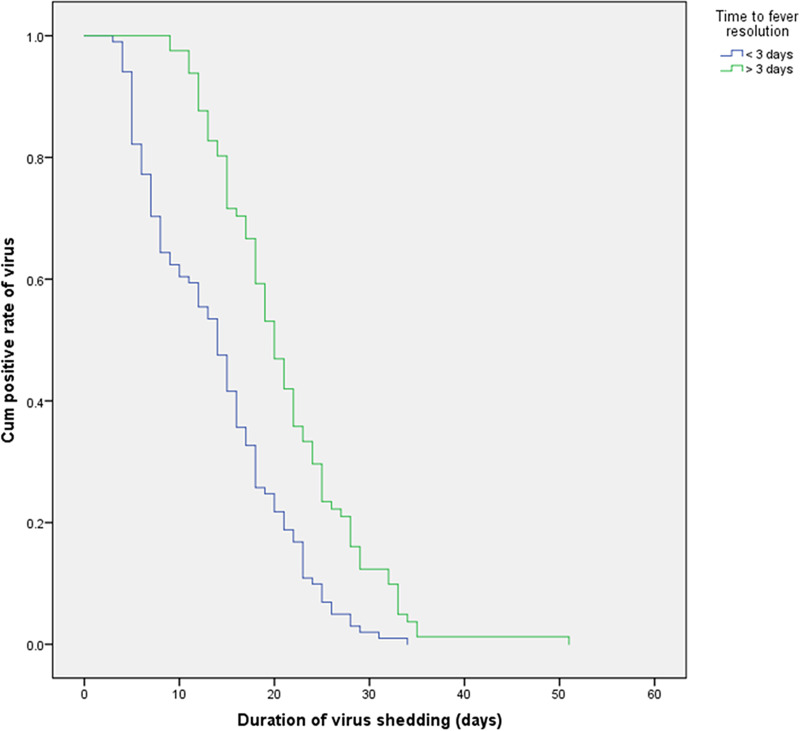
Kaplan–Meier plot for fever resolution time.

Using survival curve analysis, the viral shedding durations were compared among patients with COVID-19 with different clinical characteristics, and differences in the survival curves were analysed by the log-rank test. Kaplan–Meier analysis was used to evaluate the effects of age, comorbidities, hypoproteinaemia, bacterial pneumonia and other variables on the viral shedding duration. A Cox regression model was used to analyse the levels of CRP and CK. The results are shown in Table 5. A multivariate Cox proportional hazard model was used to analyse whether age, hypoproteinaemia, the time from symptom onset to treatment, the time to fever resolution, the presence of symptoms, treatment with corticosteroids, CRP levels and CK levels affected whether the COVID-19 patients became negative for viral nucleic acid in the treatment period (0 = no, 1 = became negative). The results showed that hypoalbuminemia (HR = 0.514; 95% CI 0.31–0.852; *P* < 0.05), a time from symptom onset to treatment >4 days (HR = 0.68; 95% CI 0.5–0.925; *P* < 0.05), a time to fever resolution >3 days (HR = 0.537; 95% CI 0.392–0.734; *P* < 0.05) and symptomatic status (HR = 0.338; 95% CI 0.189–0.605; *P* < 0.05) were independent factors influencing the viral shedding duration, as shown in Table 5. The effects on the viral shedding duration of different groups of variables in patients with COVID-19 are shown in Figures 2–5.

**Table 5.** Results of univariate analysis according to the log-rank test and multivariate analysis with a Cox proportional hazard model regarding the viral shedding duration in patients with COVID-19

Predictors	Univariate analysis				Multivariate analysis			
	HR	95% CI		*P*	HR	95% CI		*P*
		Lower	Upper			Lower	Upper	
Age	0.987	0.978	0.996	<0.001*				
Coexisting disease	0.794	0.583	1.08	0.141				
Hypoproteinaemia	0.415	0.253	0.681	<0.001*	0.514	0.31	0.852	0.01*
Bacterial pneumonia	0.8	0.583	1.099	0.169				
Time from symptom onset to treatment >4 days	0.656	0.488	0.88	0.005*	0.68	0.5	0.925	0.014*
Time to fever resolution >3 days	0.476	0.351	0.645	<0.001*	0.537	0.392	0.734	<0.001*
Symptomatic	0.221	0.127	0.387	<0.001*	0.338	0.189	0.605	<0.001*
PaO_2_/FiO_2_ on admission <400 mmHg	1.019	0.717	1.449	0.915				
CRP mg/l	0.992	0.987	0.997	0.001*				
CK U/l	0.999	0.998	1	0.007*				
Treatment with corticosteroid	0.715	0.513	0.996	0.047*				

If the variable had a *P*-value <0.05 in the univariate analysis, it was considered in the final (multivariable) model.

*P < 0.05.

## Discussion

The World Health Organization Emergency Committee for International Health Regulations declared COVID-19 a pandemic and a public health emergency of international concern. COVID-19 is an acute infectious respiratory disease, and patients with COVID-19 can worsen rapidly, progressing to acute respiratory distress syndrome (ARDS), septic shock, metabolic acidosis and coagulation dysfunction, which are difficult to treat. Huang *et al.* showed that the time from the onset of COVID-19 to the development of dyspnoea was 8 days, and that progression to ARDS occurred in 9 days [5]. Our study also found that the time from symptom onset to treatment was an independent risk factor for severe disease. A time from symptom onset to treatment >4 days was an independent factor influencing the viral shedding duration. If patients are diagnosed and treated in a timely manner, the severity of the disease can be predicted, which has important clinical significance for medical staff who are diagnosing and treating patients.

In this study, at the time of discharge, the number of patients with severe disease was 27, and the proportion of patients with severe disease had decreased to 14.59%, which was lower than the national average reported by the National Health Commission of the People's Republic of China [1] and the value reported in the study by Guan *et al*. [6]. Based on an epidemiological study of 72 314 patients with COVID-19, the mortality rate of patients with comorbidities was higher than that of patients without comorbidities, and the mortality rate of patients with diabetes was 7.3% [7]. The results showed that age and diabetes were independent risk factors for a poor prognosis. According to the current report, the proportion of COVID-19 patients with diabetes mellitus is 10.1–20%, and the proportion of critical COVID-19 patients with diabetes mellitus is 22.2% [5, 8]. The report by Klekotka *et al.* [9] pointed out that diabetes increases the risk of respiratory tract infection and is an important risk factor for the aggravation of lower respiratory tract infection. Patients with diabetes often have abnormal immune function, such as fewer immune cells and decreased NKT cell activity, rendering these patients a high-risk group for viral infections with an increased risk of severe disease [10, 11]. Diabetes leads to a chronic inflammatory response, and long-term hyperglycaemia leads to vascular endothelial cell damage, thus reducing the patient's immune status. Some studies have pointed out that the abnormal pro-inflammatory cytokine response in diabetic patients may result in severe COVID-19 [12–14]. In Mehta *et al.*'s [15] study, in patients with diabetes mellitus and COVID-19, the levels of markers such as CRP, fibrinogen and D-dimer were found to be elevated. This may be due to cytokine storms, which increase the risk of severe COVID-19 and result in a poor prognosis. Therefore, for patients with COVID-19, early diagnosis and intervention are important to reduce the risk of death caused by chronic underlying diseases such as diabetes. Age was also confirmed to be an important independent predictor of mortality in MERS [16] and SARS [17]. Zhou *et al*. reported that in-hospital death was related to age at admission [18]. With increasing age, the prognosis deteriorates, especially among the elderly population, due to the decline in immune organ function and the combination of chronic diseases. Under a certain degree of hypoxia, the heart, lung and kidney function of very elderly patients worsens, making them prone to multiple organ failure and increasing the risk of mortality [19].

The results showed that most patients with COVID-19 exhibited symptoms of fever and cough, which is consistent with the clinical signs in SARS and MERS patients [20]. A few patients had diarrhoea, but the study found that the clinical symptoms at admission could not be used to predict the severity of the disease. COVID-19 is a self-limiting disease, with most infected patients having mild cases [8]. However, in our study, more than 50% of the patients were found to have the involvement of five lung lobes on CT at admission. In a study by Shi *et al.* [21], they found lung abnormalities on CT scans in 15 patients with asymptomatic infections. Therefore, patients with symptoms should visit a physician in a timely manner and undergo CT scans. According to the inspection results, the disease severity predicted the prognosis of the patients. We found that a lower PaO_2_/FiO_2_ at the time of admission is a risk factor for a poor prognosis in patients with severe COVID-19. According to Yang *et al*. [2], the substantial difference in the PaO_2_/FiO_2_ ratio between survivors and non-survivors indicated that the PaO_2_/FiO_2_ ratio is associated with the severity of illness and prognosis. Therefore, we should pay attention to the PaO_2_/FiO_2_ index and provide respiratory support and circulatory support in a timely manner. The results showed that the time from admission to a normal temperature was 7 days for patients with severe disease and 2 days for patients with mild disease. Therefore, in the treatment of patients with COVID-19, we should pay attention to the duration of fever. For patients with a long fever duration, we should intervene in a timely manner to improve their prognosis.

The level and duration of infectious virus replication is an important factor in assessing the risk of transmission. Viral shedding is one of the most important criteria for the treatment of patients with COVID-19. Unfortunately, the pathogenesis of COVID-19 is not yet clear. SARS-CoV-2 is similar to SARS-CoV from 2003 and MERS-CoV from 2012, which mainly infect alveolar epithelial cells [22]. For patients with SARS-CoV infection, the positive rate of respiratory specimens peaked at 6–11 days after onset. More than 23 days later, respiratory specimens still showed positivity for the virus [23]. One-third of patients tested positive in respiratory specimens within 4 weeks [24]. The duration of positivity for MERS-CoV in respiratory tract samples lasted at least 3 weeks [25, 26]. In a recently published study, Zhou *et al*. [18] reported that the duration of viral shedding was 20 days. Our study showed a duration of viral shedding of 17 days, which is slightly shorter than the reported duration. It was found that the absence of hypoalbuminemia, time to fever resolution **>**3 days, time from symptom onset to treatment >4 days and symptomatic status were independent factors influencing the viral shedding duration. This finding is highly significant for the treatment of patients, the management of early-stage disease and the prevention and control of hospital infections. Hu *et al.* [27] investigated the shedding duration of SARS-CoV-2 and found that age is an independent risk factor affecting the viral shedding duration. Due to the low immune status of elderly individuals, it is more difficult for them to eradicate invasive pathogens. In our study, univariate analysis results showed that age was a risk factor for prolonged viral shedding duration, but no significant difference was found in the multivariate Cox analysis. Hypoproteinaemia can cause microcirculation disturbances and lead to insufficient perfusion of important organs and multiple organ dysfunction. Moreover, a decrease in the level of serum albumin results in decreases in the levels of various enzymes related to antibody synthesis. This decrease in enzyme activity leads to a decrease in immunity and an increase in the likelihood of infection. At the same time, hypoproteinaemia can lead to respiratory muscle atrophy, thus reducing the body's resistance and increasing the length of hospital stay and the frequency of readmission. Therefore, it takes longer for a lung infection to heal, affecting ventilation function, increasing the probability of multiple organ dysfunction and increasing mortality [28–30]. In a study in macaques, Joseph [31] found that immunosuppressed macaques exhibited significantly higher levels of MERS-CoV replication in respiratory tissues and shed more virions. Therefore, in patients with COVID-19, the timely correction of hypoproteinaemia can shorten the duration of viral shedding. Due to the limitations of this retrospective study, our study on hypoproteinaemia is lacking in detail. We will further investigate the relationship between hypoproteinaemia and viral shedding duration in future research. Since viral load detection was not carried out in the early stages, we will further study the relationship between viral load and prognosis in the future. In a study on MERS, Memish [32] noted that the time of virus clearance in asymptomatic patients was earlier than that in symptomatic patients, which is concordant with the findings reported in this study. However, we were unable to demonstrate a correlation between the duration of viral shedding and prognosis in this work. In previous studies on the shedding duration of the H7N9 virus [33], the time from symptom onset to treatment was also found to be an independent risk factor. Currently, no specific antiviral drug is available for COVID-19. We administer antiviral treatment with drugs recommended by national diagnosis and treatment guidelines [3]. Patients with mild symptoms were treated with *α*-interferon aerosol inhalation and oral umifenovir (arbidol). Umifenovir is a Russian-made small indole-derivative molecule licensed in Russia and China to prevent and treat influenza and other viral infections. For the treatment of patients with COVID-19, umifenovir is the recommended drug in Chinese diagnosis and treatment guidelines. Severe patients were treated with lopinavir or ritonavir. Approximately 90% of patients accepted treatment with a Chinese medicine decoction. Our study cannot analyse the impact factors of drugs on the viral shedding duration. However, early treatment is advantageous for improving the prognosis and shortening the viral shedding time.

All COVID-19 patients (⩾14 years) in Tianjin were included in this study. This study represents the clinical characteristics of patients in a region. This study was a retrospective study with a relatively small sample size and was performed in a single centre; however, we anticipate that our study will be of significant interest given the importance of predicting patient prognoses for this disease and promoting clinical work. One of the limitations of this study is that we assessed patients for only a limited time when they were hospitalised; thus, a longer follow-up period might be needed to further assess the prognosis of and viral shedding in cured COVID-19 patients. Future studies with longer follow-up periods and larger sample sizes and studies conducted at multiple centres are needed.

At present, no effective treatment is available for COVID-19; therefore, we analysed clinical patient data to determine the factors affecting the prognosis of the disease. This information may be used to facilitate early intervention and treatment, thereby reducing the incidence of and mortality due to severe COVID-19. This study showed that diabetes mellitus, age, the time from symptom onset to treatment and PaO_2_/FiO_2_ can predict the prognosis of patients with COVID-19. Hypoproteinaemia and the fever duration warrant special attention. Timely intervention in patients with symptoms and a time from symptom onset to treatment <4 days can shorten the duration of viral shedding.

## Data Availability

The datasets generated during and/or analysed during the current study are available from the corresponding author on reasonable request.
